# Changes of Insulin Resistance and Adipokines Following Supplementation with *Glycyrrhiza Glabra L.* Extract in Combination with a Low-Calorie Diet in Overweight and Obese Subjects: a Randomized Double Blind Clinical Trial

**DOI:** 10.15171/apb.2018.015

**Published:** 2018-03-18

**Authors:** Mohammad Alizadeh, Nazli Namazi, Elham Mirtaheri, Nafiseh Sargheini, Sorayya Kheirouri

**Affiliations:** ^1^Nutrition Research Center, Faculty of Nutrition, Tabriz University of Medical Sciences, Tabriz, Iran.; ^2^Diabetes Research Center, Endocrinology and Metabolism Clinical Sciences Institute, Tehran University of Medical Sciences, Tehran, Iran.; ^3^Molecular Biomedicine, University of Bonn, Bonn, Germany.; ^4^Department of Nutrition, Tabriz University of Medical Sciences, Tabriz, Iran.

**Keywords:** Calorie restricted diet, Licorice, Adipokine, Insulin Resistance, Obesity

## Abstract

***Purpose:*** Adipose tissue is a highly active endocrine organ which plays a key role in energy homeostasis. The aim of this study was to determine the effects of dried licorice extract along with a calorie restricted diet on body composition, insulin resistance and adipokines in overweight and obese subjects.

***Methods:*** Sixty-four overweight and obese volunteers (27 men, 37 women) were recruited into this double-blind, placebo-controlled, randomized, clinical trial. Participants were randomly allocated to the Licorice (n=32) or the placebo group (n=32), and each group received a low-calorie diet with either 1.5 g/day of Licorice extract or placebo for 8 weeks. Biochemical parameters, anthropometric indices, body composition and dietary intake were measured at baseline and at the end of the study.

***Results:*** A total of 58 subjects completed the trial. No side effects were observed following licorice supplementation. At the end of the study, waist circumference, fat mass, serum levels of vaspin, zinc-α2 glycoprotein, insulin and HOMA-IR were significantly decreased in the intervention group, but only the reduction in serum vaspin levels in the licorice group was significant when compared to the placebo group (p<0.01).

***Conclusion:*** Supplementation with dried licorice extract plus a low-calorie diet can increase vaspin levels in obese subjects. However, the anti-obesity effects of the intervention were not stronger than a low-calorie diet alone in the management of obesity.

## Introduction

Adipose tissue is a highly active endocrine organ, which plays a key role in energy homeostasis, response to hormonal signals, metabolic regulation and adipokine secretion.^1^ Current evidence suggests that adipose tissue secrets more than 50 signaling molecules and hormones, called adipokines.^1,2^Adipokines are involved in the regulation of thermogenesis, appetite, glucose metabolism, insulin sensitivity and other endocrine functions.^3^ One adipokine is vaspin, a visceral adipose tissue-derived hormone which can be considered to be a new link between obesity and metabolic complications such as insulin resistance, type 2 diabetes and atherosclerosis.^4^ Several studies have indicated an association between vaspin and body mass index (BMI); but the findings are contradictory. ^5^

Zinc-alpha 2 glycoprotein (ZAG-2) is another adipokine which plays a main role in the mobilization and utilization of lipids.^5^ It also can control fat mass (FM) and energy expenditure, induce lipolysis and act as a hormone-regulating lipid in glucose metabolism.^6^ Some studies found an inverse relationship between ZAG, body mass index (BMI) and waist circumference (WC). It has also been suggested that ZAG may simulate adiponectin to protect against inflammation and the complications of obesity.^7^

Prior studies have indicated that some medicinal herbs, such as *Nigella sativa,*^8^ green tea^9^ and *Glycyrrhiza glabra*^10,11^ are involved in the regulation of hormones and weight. *Glycyrrhiza glabra L*. (Fabaceae family), generally known as Mulaithi or Licorice, is a medicinal herb which is widely grown in the Mediterranean region and Southwest Asia. It contains various components with pharmacological properties, including glycyrrhizin, glabridin, flavonoids, beta-Glycyhrritinic acid, chalcones, isoflavones and triterpenoid saponins.^12,13^ Licorice root is frequently used in traditional medicine, particularly for gastric and duodenal ulcers, dyspepsia and allergenic reactions. Human and animal models have not demonstrated any toxic or serious side effects of licorice consumption.^14^

It has been suggested that licorice root can alter body composition^10,11,15^ and reduce insulin resistance.^16-18^ However, there are limited clinical trials with contradictory results on the effects of licorice on obesity.^10,15,19^ To the best of our knowledge, no clinical trials have evaluated the effects of licorice supplement with a low-calorie diet on the management of obesity and hormonal regulation. Accordingly, the primary aim of this study was to determine the effects of dried licorice extract together with a calorie restricted diet on anthropometric indices, body composition, insulin resistance and adipokines in overweight and obese subjects. The secondary aim was to evaluate the effects of licorice along with a low calorie diet on blood pressure and liver enzymes.

## Material and Methods

### Participants

In this double-blind randomized placebo-controlled clinical trial, 64 overweight and obese volunteers (27 men, 37 women) were recruited. Subjects were chosen by advertisement and dietitian referral from March to September 2012 at Tabriz University of Medical Sciences. A total of 64 subjects were enrolled based on FM variable in a previous study,^10^ with α-value of 0.05, power of 90% and considering a 20% loss to follow up. Inclusion criteria were as follows: age 30-60 yrs old and BMI>25 kg/m.^2^ The following were used as exclusion criteria cardiovascular disease, liver, thyroid and kidney disorders, diabetes, smoking, pregnancy or lactation of having taken any anti-obesity, vitamin and mineral supplements or herbal drugs in the 3 months prior to the study. Subjects who consumed any medications for hypertension or had Systolic Blood Pressure (SBP) ≥140 mmHg and Diastolic Blood Pressure (DBP) ≥ 90 mmHg were also excluded.

At the beginning of the trial, general characteristics including age, medication history and any family history of obesity were collected using a questionnaire.

### Study Design and Intervention

Eligible participants were randomly allocated to the licorice (n=32) or placebo groups (n=32). Randomization was facilitated by random number table with a permuted block size of two. The participants were stratified for sex, age and BMI. All of the participants received a low-calorie diet; created by an expert dietitian who designed individualized diets with a 500 kcal deficit from the participates' energy requirements. The calories provided by the diets consisted of 55% carbohydrate, 15% protein and 30%. The intervention and placebo groups took 0.5 g/day (3 times a day 30 min before each meal) of dried licorice extract and placebo (corn starch), respectively for 8 consecutive weeks. To maintain blinding, a subject with no clinical involvement in the study performed the allocation. The patients and investigators remained blinded to the treatment assignment until data analysis. Visits occurred every 20 days and supplements were distributed among the volunteers based on the allocation code after the randomization. Participants received a phone call every week to minimize withdrawal and ensure their adherence to the study protocol. The subjects were asked to maintain their usual physical activity level during the trial.

### Licorice extract characteristics

The dried hydroalcoholic extract of licorice root (ethanol 70: water 30% v/v) was prepared by the Darook pharmacological company (Esfahan-Iran). It contained lowered Glycyrrhizin (<0.01%).

### Measurements

Anthropometric indices, body composition, dietary intake, physical activity, blood pressure and biochemical parameters were measured at baseline and at the end of the study. Weight, height and WC were measured using standard methods. Assessment of dietary intake and physical activity levels were measured as explained in our previous study.^12^

#### Body composition measurements

BMI was calculated by dividing the weight in kilograms to the square of the height in meters. Body composition was measured using TANITA Bioelectrical Impedance Analysis (BC-418 MA, 50 kHz) after 12-14 hours fasting‏. We measured the amounts of FM and fat free mass (FFM) with an accuracy of ±0.1 kg. Previous studies reported a significant correlation between TANITA and Dual Energy X-Ray absorptiometry (DEXA) test for the measurement of body composition.

#### Biochemical measurements

At the baseline and at the end of the trial, 10 mL of venous blood was collected after 12-14h fasting. The serum was separated from whole blood by centrifugation at 2500 rpm for 10 min. Serum levels of Fasting blood sugar (FBS) was measured on the day of sampling using Auto analyzer (Abbot Model Aclyon 300 USA) by commercially enzymatic kit (Pars Azmoon, Iran). The remaining serum samples were kept at -20 °C until measurement. Enzyme-linked immunosorbent assay (ELISA) method was used to determine insulin (Pars Azmoon, Iran), vaspin (Orgenium, Fenland) and ZAG-alpha 2 (Orgenium, Fenland) concentrations. Based on FBS and insulin levels, insulin resistance was evaluated using the homeostasis model assessment-insulin resistance (HOMA-IR) formula as follows:

HOMA-IR = fasting glucose (mg/dL) ×fasting insulin (μU/mL) /405^20^

#### Blood pressure measurement

Blood pressure was measured after 10 minutes rest in seated and relaxed position using a Microlife AG-30 mercury sphygmomanometer on the left arm. It was repeated after 5 min and the average of the two measurements was reported.

### Statistical analysis 

Data were analyzed using SPSS software version 13.0 (SPSS Inc., Chicago, IL, USA). The normality of the data distribution was evaluated by the one-sample Kolmogorov-Smirnov test. The results were expressed as mean±SD for variables with normal distribution, median (25^th^, 75^th^ percentiles) for variables with non-normal distribution, and percentage (%) for qualitative variables. The Chi square test was used for the comparison of qualitative variables. Independent *t* tests (for baseline measurements) and analysis of covariance (ANCOVA) were used to compare quantitative variables between groups, controlling for confounding factors. The Mann-Whitney U test was used for comparison variables with non-normal distribution between two groups. Pair t-test was also used for within- group comparison. p<0.05 was considered statistically significant.

## Results

**Figure 1:**
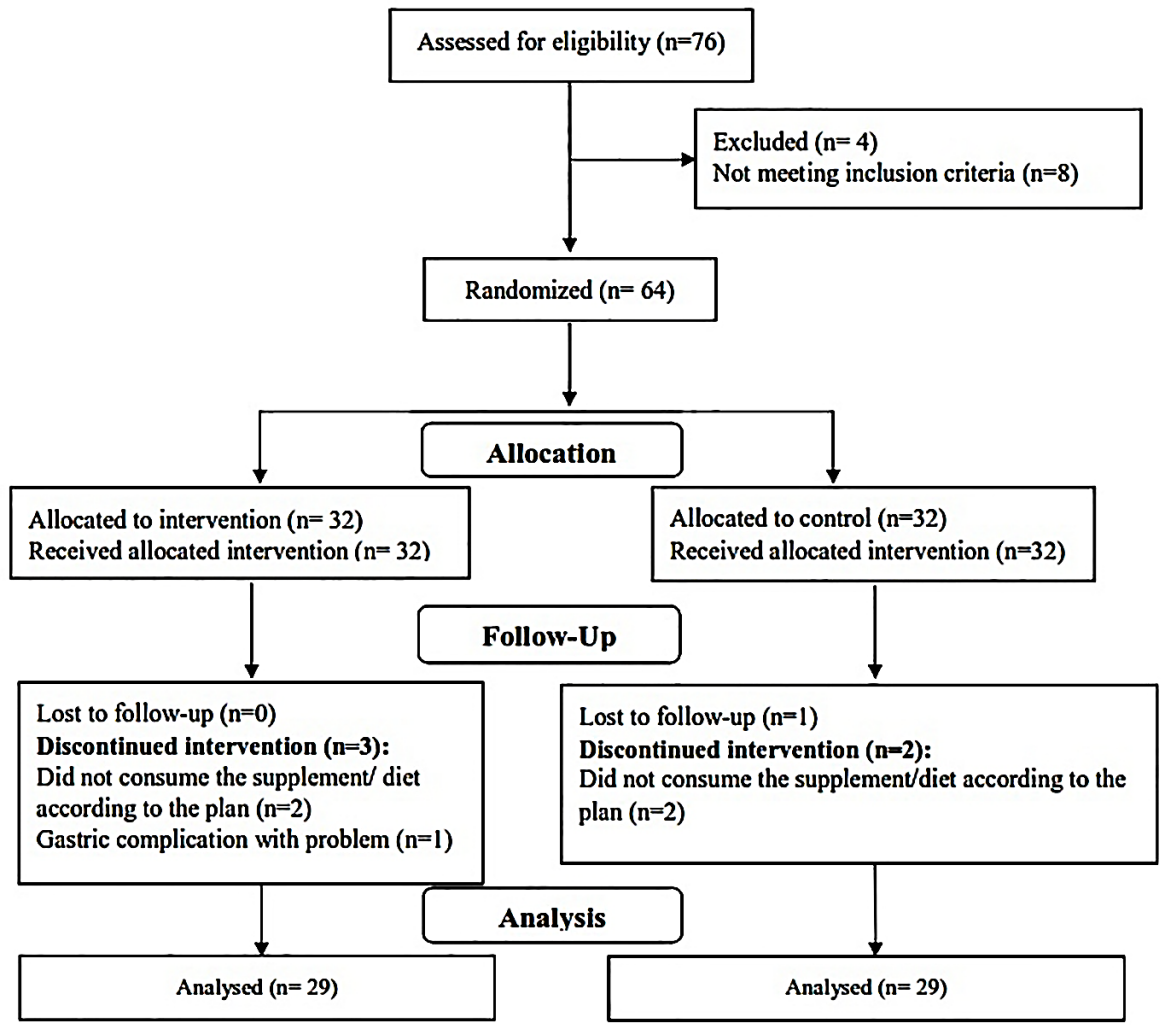
Flowchart of the study

As presented in Figure 1, of the 64 participants, 58 subjects completed the study (intervention group, n=29; placebo group, n=29). The power of the study at the end of the study was 85%. Participants did not report any serious side effects for taking licorice supplement, except one who reported gastrointestinal problems and discontinued the study.

There were no significant differences between the two study groups (except in height) at the baseline (Table 1). Table 3 shows anthropometric indices and body composition at baseline and at the end of the study. No significant differences were observed in the licorice and placebo groups at the start of the study. In the licorice group, a slight reduction (-2.3%) was observed in BMI at the end of the trial, but it was not significant within or between the groups (ANCOVA; adjusted for height and baseline value). FM decreased significantly at the end of the study in both groups when compared to the baseline (-7.2 vs. -6.5%; p<0.01). However, a comparison of licorice and placebo groups did not indicate any significant reduction in FM after the intervention (p=0.6). In both the licorice and placebo groups, FFM slightly increased (0.3%; p=0.8) and significantly decreased (-1.3%; p<0.01), respectively, but inter group comparisons did not show any significant differences in FFM at the end of the trial (p=0.7).

**Table 1:** Baseline characteristics of the study participants

**-**	**Variables**	**Licorice group (n=29)**	**Placebo group (n=29)**
	Age (year)	36.0 ± 11.9*	33.6 ± 4.8
Sex (n(%))	Male	13 (44.8)	14 (48.2)
	Female	16 (55.2)	15 (51.8)
-	Weight(kg)	87.6 ± 15.5	81.9 ± 11.0
	Height(cm)	161.9 ± 8.3	158.4 ± 5.8
Physical activity (n(%))	Sedentary	18 (62.0)	16 (55.1)
	Moderate	11 (38.0)	13 (44.9)

* Mean± SD

No adverse effects were observed on blood pressure (Table 2) and biochemical tests (Table 3) (p>0.05 for all variables). In this present study, the licorice supplement contained less than 0.01% Glycyrrhizin, so no significant changes were observed in SBP and DBP after 8 weeks of the intervention.

Biochemical parameters are presented in Table 3. At baseline there were no significant differences between the two study groups in biochemical parameters, except in fasting blood sugar (FBS) levels. Serum levels of FBS was not affected by the intervention (p=0.8). However, comparison between the two groups indicated that, insulin concentrations and HOMA-IR decreased in both the groups after 8 weeks of the intervention, and that significant reductions in insulin and insulin resistance were only observed in the licorice group when compared to baseline (ANCOVA, adjusted for changes in weight, energy intake changes, and baseline values). Further, no significant reduction of the two factors were observed between the two groups (p<0.05 for both variables).

Furthermore, levels of fat-derived hormones (vaspin and ZAG) also changed significantly following the licorice supplementation plus weight-loss diet when compared to baseline (-27.8 and 32.2%, respectively). Comparison of the licorice and placebo groups indicated that only the changes in serum levels of vaspin was significant at the end of the study (-27.8 vs. -4.2%, respectively).

**Table 2:** Comparison of anthropometric indices and body composition between Licorice and placebo groups at baseline and at the end of the trial

**-**	**Variable**	**Licorice group (n=29)**	**Placebo group (n=29)**	P-value** **(Between groups)**
**BMI (Kg/m** ^ 2 ^ **)**	Baseline	33.6±4.8*	32.7±3.7	0.4†
	End	32.8±4.8	32.3±3.5	0.2‡‡
	Pre to post P-value‡	0.3	0.6	
**Waist circumference (cm)**	Baseline	106.9±13.4	108.9±10.4	0.5†
	End	101.3±10.9	102.4±10.1	0.7
	Pre to post P-value‡	<0.01	<0.01	-
**FM (Kg)**	Baseline	31.7±8.3	30.6±6.7	0.5†
	End	29.4±10.6	28.6±6.3	0.6
	Pre to post P-value‡	<0.01	<0.01	-
**FFM (Kg)**	Baseline	55.9±10.7	51.3±8.0	0.04†
	End	56.1±12.0	50.6±5.1	0.7
	Pre to post P-value‡	0.8	<0.01	-
**SBP (mmHg)**	Baseline	110.2±10.1	110.5±10.5	0.4†
	End	109.0±10.0	110.0±10.4	0.8
	Pre to post P-value‡	0.2	0.2	-
**DBP (mmHg)**	Baseline	70.3±7.0	70.3±8.0	0.9†
	End	70.1±9.0	70.2±10.0	0.4
	Pre to post P-value‡	0.2	0.8	-

FM: Fat Mass; FFM: Fat Free Mass; SBP: Systolic Blood Pressure; DBP: Diastolic Blood Pressure * Mean± SD ** ANCOVA (adjusted for energy intake changes and baseline values) † Independent t-test ‡ Paired t-test ‡‡ ANCOVA (adjusted for height and baseline values)

## Discussion

The present study highlights that licorice extract supplementation concurrently with a low-calorie diet, sufficiently attenuates serum levels of vaspin hormone in overweight and obese subjects with no significant side effects. The findings also revealed that a low-calorie while taking licorice supplementation was no more efficacious than a low-calorie diet alone on the management of obesity.

**Table 3:** Comparison of biochemical parameters between Licorice group and placebo group at baseline and at the end of trial

**-**	**Variable**	**Licorice group (n=29)**	**Placebo group (n=29)**	P-value (Between groups)**
**FBS (mg/dL)**	Baseline	98.5± 7.8*	93.2 ± 7.1	0.02†
	End	97.5 ± 7.3	93.5± 6.5	0.8
	**Pre to post P-value**‡	0.4	0.7	-
**Vaspin (ng/mL)**	Baseline	24.4±9.3	21.1±4.9	0.13†
	End	17.6±3.7	20.2±5.4	<0.01
	**Pre to post P-value**‡	0.01	0.1	-
**ZAG (µg/mL)**	Baseline	86.1±40.0	92.3±34.8	-
	End	113.9±57.5	101.3±40.2	0.5†
	**Pre to post P-value**‡	<0.01	0.4	0.4
**Insulin (µU/mL)**	Baseline	9.5 (5.3, 12.2)	9.5 (7.2, 13.5)	0.7†
	End	7.1 (5.0, 8.5)	9.2 (4.7, 11.0)	0.2
	**Pre to post P-value**‡	0.02	0.1	-
**HOMA-IR**	Baseline	2.3 (1.3, 3.0)	2.2 (1.3, 3.0)	0.8‡‡
	End	1.5 (1.2, 2.0)	2.0 (1.1, 2.5)	0.3
	**Pre to post P-value**‡	<0.01	0.4	-
**AST** (U/L)	Baseline	16.6±3.9	17.8±4.0	0.09†
	End	16.0±2.4	17.0±5.3	0.07
	**Pre to post P-value**‡	0.4	0.7	-
**ALT** (U/L)	Baseline	15.5±2.7	16.6±7.8	0.2†
	End	15.0±6.4	16.0±7.5	0.3
	**Pre to post P-value**‡	0.7	0.9	-

FBS: Fasting blood sugar; ZAG: Zinc alpha2 glyco protein; AST: Aspartate transaminase; ALT:Alanine aminotransferase * Mean± SD ** ANCOVA (adjusted for weight changes, energy intake changes and baseline values) † Independent t-test for variables with normal distribution and Mann-Whitney U test for variables with non-normal distribution ‡ Paired t-test ‡‡ ANCOVA (adjusted for FBS at baseline)

There are limited clinical trials with contradictory findings on the effects of licorice supplementation on anthropometric indices and body composition. Our findings were in accordance with two studies; Bell *et al.* reported that Glavonoid^TM^(Licorice Flavenoid Oil (LFO)) did not reduce body weight, FM and WC in overweight and grade I-II obese subjects after 8 weeks.^19^ Moreover, Hajiaghamohammadi *et al.* reported that 2 g/day aqueous licorice extract did not reduce BMI in patients with non-alcoholic fatty liver disease after 8 weeks.^21^ Our results were in opposition to Tominaga *et al.'s* study; who found that 300 and 1800 mg/day supplementation with Kaneka Glavonoid rich oil ^TM^ (LFO) suppressed weight gain in overweight subjects with unhealthy lifestyle after 12 weeks. However, weight and BMI was increased at the end of the study in the placebo group. In addition, supplementation with 900 mg/day LFO decreased visceral fat in overweight subjects. They suggested that the reduction in FM was helpful in weight maintenance.^10^ In another study in the U.S population, Tominaga *et al.* indicated that 300 mg/day LFO decreased WC and visceral fat after 12 weeks.^22^

Based on Armanina *et al's.,* study, 3.5 g/day Licorice supplement decreased FM with no changes in BMI in normal weight subjects after 8 weeks.^15^ Aoki *et al.* also indicated that adding 1 and 2% LFO to diet of obese mice for 8 weeks significantly slowed down weight gain and decreased abdominal white adipose tissue.^11^ Differences in results of these studies may be due to differences in energy intake, physical activity level, dose and type of licorice (extract, oil), the duration of the intervention, BMI range and ethnic group. In our study, we compared the efficacy of licorice supplement concurrent with a calorie-restricted diet vs. a calorie-restricted diet alone. Based on evidence, differences between two groups were not significant at the end of the trial. It seems that licorice plus a weight loss diet prevents a reduction in FFM. However, they were not significant between the two intervention groups.

In this study, licorice extract with a calorie-restricted diet did not decrease serum levels of FBS, insulin concentrations, ZAG and HOMA-IR; but the intervention decreased serum levels of vaspin after 8 weeks. Limited clinical trials have evaluated the effects of licorice on glycemic status. In line with our study, Tominaga *et al.* found that 1800 mg/day LFO did not change FBS and insulin concentrations in overweight subjects after 12 weeks.^10^ However, Luan *et al.* indicated that 10 µMg of glabridin, a main component of licorice, decreased insulin levels and insulin resistance in women with polycystic ovary syndrome after 12 months.^16^ Zhao *et al.* demonstrated that 300 mg/day of licorice flavonoid decreased FBS and insulin levels in type 2 diabetic rats after 5 weeks,^17^ and on Wu *et al.'s* study demonstrated that 40 mg/kg/day glabridin decreased FBS and insulin resistance in diabetic mice after 28 days.^18^

In ourstudy, as no significant changes were observed in serum levels of FBS and insulin, insulin resistance did not change following the supplementation with Licorice extract. Reduction in body weight and fat mass are two main factors involve in improving insulin resistance in overweight and obese subjects. However, Licorice extract did not reduce these two anthropometric indices considerably. Therefore, no changes in insulin resistance might be due to this issue. In the current trial, we used the index of HOMA-IR to examine insulin resistance. There are several indices to assess this parameter.^23^ Using different indices based on their different components can affect the results. Moreover, observing no changes in insulin resistance can be partially explained by the type of supplement. The aforementioned studies have examined licorice flavonoid, while our study assessed whole licorice extract. Different dosages, types of supplement, amounts of flavonoid, changes in weight, BMI at baseline, duration of the intervention, and methods for insulin resistant estimation are possible factors that can lead to different findings.

In our study, due to mineralcorticoid actions and vasopresser effects of Glycyrrihizin,^24,25^ Glycyrrihizinhas been reduced to <0.01%. This could be attributed to the observation that the supplementation did not lead to any significant reduction in FBS and insulin concentrations. Furthermore, patient medical history, baseline BMI, dosages and form of licorice or its pure component and the duration of intervention can affect the final findings of each study.

To the best of our knowledge, our study was the first to evaluate the effects of supplementation with licorice extract on vaspin and ZAG hormone levels. Vaspin is an adipokine with insulin- sensitizing effects, and may be involved in obesity-associated diseases including type 2 diabetes, insulin resistance, atherosclerosis and cardiovascular disease. Therefore, it may be a possible target in the pharmaco-therapeutic treatment of obesity and its complications.^5,26^ Handisurya *et al.* reported that weight loss following gastric bypass decreased BMI and vaspin hormones in morbidly obese subjects after 12 months. They declared that visceral adipose tissue was the predominant localization for vaspin gene expression, and vaspin secretion decreased due to significant reductions in BMI after gastric bypass.^27^ Based on Chung *et al,.* study lifestyle modification with orlistat decreased BMI and vaspin levels after 12 weeks. They hypothesized that weight loss (BMI reduction ≥ 2%) leads to a reduction in vaspin levels.^28^ In this study, it seems that a larger reduction in BMI (-2.3%) in the licorice group compared to the placebo group might have resulted in a reduction in vaspin levels. However, due to the absence of adequate studies on the effects of licorice on vaspin levels, the underlying mechanisms are not clear. The results of our study contradict those of Koiuo *et al.* regarding changes in vaspin. They found that a calorie-restricted diet with orlistat or sibutramine decreased weight with no changes in vaspin concentrations after 6 months.^29^ This discrepancy could be due to differences in the type of intervention, study duration and the rate of weight and BMI reduction.

Zinc-alpha 2 glycoprotein is an adipokine secreted from white and brown adipose tissues. Some studies have reported its possible effects on obesity and metabolic syndrome.^5,6^The expression of ZAG is regulated by TNF-alpha and PPAR-γ; thus, it may participate in lipid metabolism, enhance energy expenditure and skeletal muscle glucose transporters, inhibit of enzymes in lipogenesis pathways and stimulate adiponectin hormone expression.^30^ In this study, there were no significant differences between the two groups. It seems that a greater reduction in weight or FM is needed to change levels of ZAG.

The main side effects of licorice are hypertension and hypokalemic-induced secondary disorders.^31^ Glycyrrhizin plays a key role in occurring these side effects.^31^ Therefore, in the present study we used a kind of licorice extract with reduced glycyrrhizin. Accordingly, except one who reported stomachache no side effects were reported.

This had some limitations. Firstly, the effects of licorice supplementation without calorie restricted diet were not evaluated. Secondly, the duration of the intervention was short and thirdly gene expressions of hormones were not measured. For future studies, we suggest that higher dosages and different forms (oil, pure components of licorice such as glabridin and flavonoids) of licorice extract are evaluated for their effect on the management of obesity.

## Conclusion

We conclude that supplementation with dried licorice extract plus a low-calorie diet can increase vaspin levels in obese subjects, with no changes in insulin resistance and body composition. Overall, the effects of the intervention was not stronger than a low-calorie diet alone in the management of obesity.

## Acknowledgments

We are grateful to the participants for their cooperation. The authors also would like to thank the Nutrition Research Center, Tabriz University of Medical Sciences for funding the project.

## Ethical Issues

The trial was approved by the Ethics Committee of Tabriz University of Medical Sciences and written informed consent was obtained from each patient. The trial was registered on the Iranian registry of clinical trials (www.irct.ir/, IRCT2013062811288N3).

## Conflicts of Interest

Authors declare no conflicts of interest

## References

[1] Galic S, Oakhill JS, Steinberg GR (2010). Adipose tissue as an endocrine organ. Mol Cell Endocrinol.

[2] Bluher M (2009). Adipose tissue dysfunction in obesity. Exp Clin Endocrinol Diabetes.

[3] Poulos SP, Hausman DB, Hausman GJ (2010). The development and endocrine functions of adipose tissue. Mol Cell Endocrinol.

[4] Auguet T, Quintero Y, Riesco D, Morancho B, Terra X, Crescenti A (2011). New adipokines vaspin and omentin. Circulating levels and gene expression in adipose tissue from morbidly obese women. BMC Med Genet.

[5] Bluher M (2012). Vaspin in obesity and diabetes: Pathophysiological and clinical significance. Endocrine.

[6] Gong FY, Zhang SJ, Deng JY, Zhu HJ, Pan H, Li NS (2009). Zinc-alpha2-glycoprotein is involved in regulation of body weight through inhibition of lipogenic enzymes in adipose tissue. Int J Obes (Lond).

[7] Cabassi A, Tedeschi S (2013). Zinc-alpha2-glycoprotein as a marker of fat catabolism in humans. Curr Opin Clin Nutr Metab Care.

[8] Mahdavi R, Alizadeh M, Namazi N, Farajnia S (2016). Changes of body composition and circulating adipokines in response to nigella sativa oil with a calorie restricted diet in obese women. J Herb Med.

[9] Chan CC, Koo MW, Ng EH, Tang OS, Yeung WS, Ho PC (2006). Effects of chinese green tea on weight, and hormonal and biochemical profiles in obese patients with polycystic ovary syndrome--a randomized placebo-controlled trial. J Soc Gynecol Investig.

[10] Tominaga Y, Mae T, Kitano M, Sakamoto Y, Ikematsu H, Nakagawa K (2006). Licorice flavonoid oil effects body weight loss by reduction of body fat mass in overweight subjects. J Health Sci.

[11] Aoki F, Honda S, Kishida H, Kitano M, Arai N, Tanaka H (2007). Suppression by licorice flavonoids of abdominal fat accumulation and body weight gain in high-fat diet-induced obese c57bl/6j mice. Biosci Biotechnol Biochem.

[12] Mirtaheri E, Namazi N, Alizadeh M, Sargheini N, Karimi S (2015). Effects of dried licorice extract with low-calorie diet on lipid profile and atherogenic indices in overweight and obese subjects: A randomized controlled clinical trial. Eur J Integr Med.

[13] Ahn J, Lee H, Jang J, Kim S, Ha T (2013). Anti-obesity effects of glabridin-rich supercritical carbon dioxide extract of licorice in high-fat-fed obese mice. Food Chem Toxicol.

[14] Parvaiz M, Hussain K, Khalid S, Hussnain N, Iram N, Hussain Z (2014). A review: Medicinal importance of glycyrrhiza glabra L.(fabaceae family). Global J Pharmacol.

[15] Armanini D, De Palo CB, Mattarello MJ, Spinella P, Zaccaria M, Ermolao A (2003). Effect of licorice on the reduction of body fat mass in healthy subjects. J Endocrinol Invest.

[16] Luan B-G, Sun C-X (2015). Effect of glabridinon on insulin resistance, c-reactive protein and endothelial function in young women with polycystic ovary syndrome. Bangladesh J Pharmacol.

[17] Zhao H, Wang Y, Wu L, Yongping MA (2012). Effect of licorice flavonoids on blood glucose, blood lipid and other biochemical indicators in type 2 diabetic rats. China J Physiol.

[18] Wu F, Jin Z, Jin J (2013). Hypoglycemic effects of glabridin, a polyphenolic flavonoid from licorice, in an animal model of diabetes mellitus. Mol Med Rep.

[19] Bell ZW, Canale RE, Bloomer RJ (2011). A dual investigation of the effect of dietary supplementation with licorice flavonoid oil on anthropometric and biochemical markers of health and adiposity. Lipids Health Dis.

[20] Dai CY, Huang JF, Hsieh MY, Hou NJ, Lin ZY, Chen SC (2009). Insulin resistance predicts response to peginterferon-alpha/ribavirin combination therapy in chronic hepatitis c patients. J Hepatol.

[21] Hajiaghamohammadi AA, Ziaee A, Samimi R (2012). The efficacy of licorice root extract in decreasing transaminase activities in non-alcoholic fatty liver disease: A randomized controlled clinical trial. Phytother Res.

[22] Tominaga Y, Kitano M, Mae T, Kakimoto S, Nakagawa K (2014). Effect of licorice flavonoid oil on visceral fat in obese subjects in the united states. Nutrafoods.

[23] Park SE, Park CY, Sweeney G (2015). Biomarkers of insulin sensitivity and insulin resistance: Past, present and future. Crit Rev Clin Lab Sci.

[24] Zadeh JB, Kor ZM, Goftar MK (2013). Licorice (glycyrrhiza glabra linn) as a valuable medicinal plant. Int J Adv Biol Biom Res.

[25] Shimoyama Y, Hirabayashi K, Matsumoto H, Sato T, Shibata S, Inoue H (2003). Effects of glycyrrhetinic acid derivatives on hepatic and renal 11beta-hydroxysteroid dehydrogenase activities in rats. J Pharm Pharmacol.

[26] Russell ST, Tisdale MJ (2010). Antidiabetic properties of zinc-alpha2-glycoprotein in ob/ob mice. Endocrinology.

[27] Handisurya A, Riedl M, Vila G, Maier C, Clodi M, Prikoszovich T (2010). Serum vaspin concentrations in relation to insulin sensitivity following rygb-induced weight loss. Obes Surg.

[28] Chung HK, Chae JS, Hyun YJ, Paik JK, Kim JY, Jang Y (2009). Influence of adiponectin gene polymorphisms on adiponectin level and insulin resistance index in response to dietary intervention in overweight-obese patients with impaired fasting glucose or newly diagnosed type 2 diabetes. Diabetes care.

[29] Koiou E, Tziomalos K, Dinas K, Katsikis I, Kalaitzakis E, Delkos D (2011). The effect of weight loss and treatment with metformin on serum vaspin levels in women with polycystic ovary syndrome. Endocr J.

[30] Stejskal D, Karpisek M, Reutova H, Stejskal P, Kotolova H, Kollar P (2008). Determination of serum zinc-alpha-2-glycoprotein in patients with metabolic syndrome by a new elisa. Clin Biochem.

[31] Nazari S, Rameshrad M, Hosseinzadeh H (2017). Toxicological effects of glycyrrhiza glabra (licorice): A review. Phytother Res.

